# Strategies to increase catch-up vaccination among migrants: a qualitative study and rapid review

**DOI:** 10.1093/eurpub/ckac131.116

**Published:** 2022-10-25

**Authors:** A Deal, AC Crawshaw, M Salloum, SE Hayward, F Knights, LP Goldsmith, J Carter, K Rustage, S Mounier-Jack, S Hargreaves

**Affiliations:** 1 Institute for Infection and Immunity, St George’s, University of London, London, UK; 2 Faculty of Public Health and Policy, LSHTM, London, UK

## Abstract

**Background:**

WHO’s Immunization Agenda 2030 has placed renewed focus on catch-up vaccination across the life course to meet global targets for reduction in vaccine-preventable diseases through increased vaccine coverage, including among migrant groups who may require catch-up vaccination to align them with host country vaccination schedules.

**Methods:**

We did a global rapid review (01/2010 to 04/2022) to explore drivers of vaccine hesitancy among migrants followed by an in-depth qualitative study (semi-structured, telephone interviews) among recently arrived adult migrants (foreign-born, >18 years old, residing in the UK < 10 years). Interviews explored views on routine vaccination including accessibility, confidence and awareness. Data were analysed iteratively using thematic analysis.

**Results:**

63 papers were included in the rapid review, including data from 22 countries/regions. Multiple factors driving under-immunisation and hesitancy in migrants were reported, including language barriers, low health literacy, social exclusion, low cultural competency and accessibility in healthcare systems. Our qualitative study recruited 40 migrants (mean age: 36.7 years; 62.5% female) resident in the UK (6 refugees, 19 asylum-seekers, 8 undocumented, 7 labour migrants). Major barriers to catch-up vaccination included a lack of provider recommendation and low awareness, with vaccination viewed as only relevant to children. Hesitancy around specific vaccines, such as MMR, was often influenced by misinformation. Participants suggested that novel strategies such as walk-in or mobile access points, consistent provider recommendations, and translation of information into relevant languages, may enhance accessibility and uptake of routine vaccinations.

**Conclusions:**

Targeted and tailored information campaigns, versatile and proactive access pathways and education for healthcare staff on cultural competency will be needed to ensure uptake of catch-up vaccination among marginalised migrant groups.

**Key messages:**

• Newly arrived adult migrants face barriers to catch-up vaccination in host countries, which may hinder immunisation coverage and increase the risk of vaccine-preventable disease outbreaks.

• Health systems must develop novel mechanisms to proactively offer culturally competent and accessible catch-up vaccination services to adult migrants on and after arrival.

